# Hyperreflective photonic crystals created by shearing colloidal dispersions at ultrahigh volume fraction

**DOI:** 10.1038/s41378-024-00651-2

**Published:** 2024-01-31

**Authors:** Minji Kim, Jong Bin Kim, Shin-Hyun Kim

**Affiliations:** Department of Chemical and Biomolecular Engineering, Korea Advanced Institute of Science and Technology (KAIST), Daejeon, 34141 Republic of Korea

**Keywords:** Nanoparticles, Nanoparticles

## Abstract

Colloidal crystallization serves as one of the most economic and scalable production methods for photonic crystals. However, insufficient optical performance, nonuniformity and low reproducibility remain challenges for advanced high-value applications. In this study, we optimally formulate a photocurable dispersion of silica particles and apply shear flow to unify the orientation of the colloidal crystals, ensuring high optical performance and uniformity. The silica particles experience strong repulsion at ultrahigh volume fractions of 50% but demonstrate low mobility, leading to polycrystalline structures. Applying shear flow to the dispersions allows the silica particles to rearrange into larger crystalline domains with a unidirectional orientation along the flow. This shear-induced structural change produces absolute reflectivity at the stopband as high as 90% and a high transparency of 90% at off-resonant wavelengths with minimal diffusive scattering. Furthermore, the strong interparticle repulsion ensures a uniform volume fraction of particles throughout the dispersion, reducing deviations in the optical properties. We intricately micropattern the photocurable dispersions using photolithography. Additionally, the photonic films and patterns can be stacked to form multiple layers, displaying mixed structural colors and multiple reflectance peaks without sacrificing reflectivity. These superior photonic materials hold promise for various optical applications, including optical components and anticounterfeiting patches.

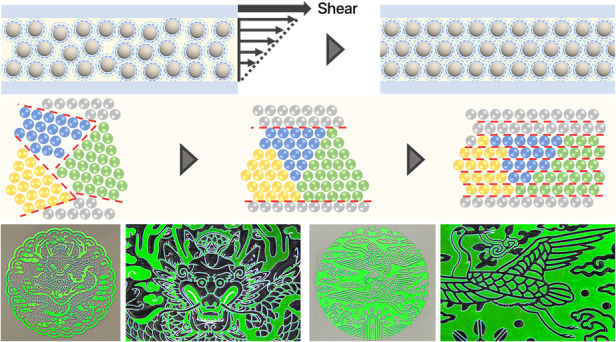

## Introduction

In nature, periodic nanostructures produce physically originated colors, known as structural colors, which play a crucial role in the survival of creatures^1–3^. In plants, structural colors serve various purposes, such as attracting insects and birds, defending against herbivores, providing protection from ultraviolet light, and enhancing the capture of photosynthetically important wavelengths^4,5^. Structural colors are also prevalent in animals, from the smallest insects and arachnids to birds and chameleons, where they are used for camouflage, mating selection, and warning predators^6,7^. Inspired by nature, periodic nanostructures have been artificially created to exhibit colors that are intensely vivid, tunable, resistant to fading, and nontoxic. The self-assembly of monodisperse colloidal particles into regular lattices provides a relatively simple and cost-effective approach for generating photonic nanostructures^8–12^. The periodic arrangement of these colloidal particles, known as colloidal crystals, induces wavelength-selective diffraction within the stopband, leading to the development of structural colors. By adjusting the lattice constant or the effective refractive index, the color or diffraction wavelength can be controlled^13–18^.

Various strategies for creating colloidal crystals have been proposed, but they have some limitations for commercial applications^16^. The popular method of evaporation-induced self-assembly produces high-quality colloidal crystals but suffers from low throughput and poor mechanical stability^19–21^. Spray coating provides a simple and easy method, but its optical performance is significantly limited due to the low crystallinity of the assembly^22–24^. One of the most pragmatic methods for producing colloidal photonic crystals involves the use of photocurable dispersions of silica particles in acrylate-based resins^10,14,25–28^. The silica particles possess a solvation layer on their surfaces, providing their repulsive interparticle potential and leading to evaporation-free spontaneous crystallization at high volume fractions. Furthermore, the photopolymerization of resins enables the instant capture of the crystalline arrays for the production of various formats, such as films, micropatterns, and microparticles, in conjunction with microfabrication techniques, such as film casting, photolithography, micromolding, and microfluidics^14,20,29–38^. This method provides ease of processing, high mechanical stability, and an acceptable level of optical performance for various coloration purposes^39–44^. However, their optical performance needs to be improved and their uniformity and reproducibility for advanced high-value applications, such as optical components, need to be ensured. This remains an important challenge and necessitates a systematic study of various parameters and conditions.

In this study, we revisit photocurable dispersions to achieve superior optical performance through volume fraction optimization and shear-assisted crystallization. We significantly enhance the uniformity and reproducibility, overcoming long-standing obstacles for applications. The key to our success is the shear-induced reorganization of colloidal particles at an ultrahigh volume fraction of 50%. Although shear-induced crystallization has been investigated for various colloidal systems, including aqueous suspensions and fused core-shell particles^36,45^, no systematic study on the shear-induced crystallization of silica particles dispersed in acrylate-based resin has been performed. Interparticle repulsion strengthens with the particle volume fraction in the presence of a solvation layer as the interparticle separation decreases. While this repulsion generally promotes spontaneous crystallization, strong repulsion and limited mobility of particles result in polycrystalline structures composed of small domains. Shearing the dispersions at an ultrahigh volume fraction facilitates particle rearrangement, which redirects the crystal orientation along the shear, thereby producing unidirectional crystals. In contrast, particles at lower volume fractions of 30% and 40% have relatively high mobility but experience weaker repulsion and yield low crystallinity even under shear flow. With the shear condition optimized for ultrahigh volume fraction, we achieve an absolute reflectivity at the stopband as high as 90% and an average transparency of 90% at off-resonant wavelengths due to high crystallinity. Interestingly, the optimum conditions occur at the rheological transition from the linear viscous region to the loss-modulus overshoot region rather than the loss-modulus overshoot region. Since the resulting photonic films are highly transparent, we can stack multiple films to develop multiple peaks in the reflectance spectra without compromising their reflectivity. Additionally, these multiple peaks produce mixed structural colors, which are highly uniform across entire areas with a high uniformity of all films. We believe that these single and multistacked photonic crystal films with high reflectivity have potential for a variety of applications, such as optical filters, reflectors, and anticounterfeiting optical barcodes as well as structural colorants and colorimetric sensors^10,40–43^.

## Results and Discussion

### Influence of the particle volume fraction

**Fig. 1:**
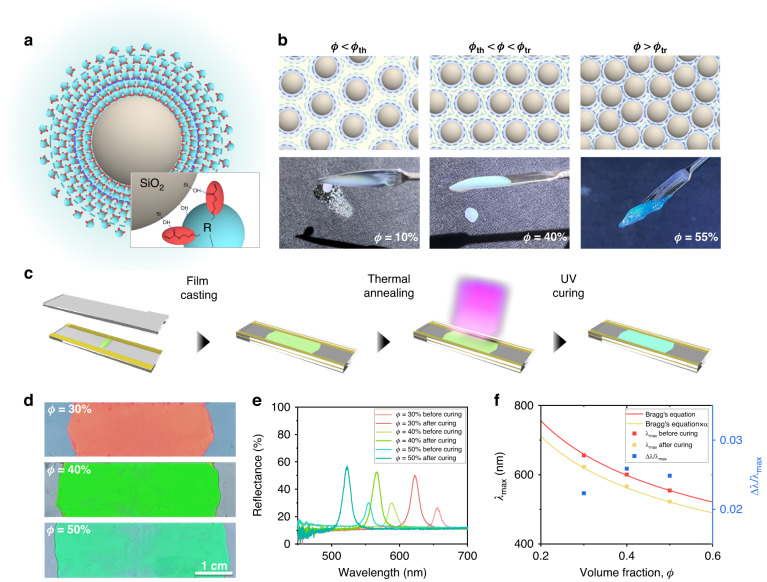
Influence of the particle volume fraction. **a** Scheme showing the formation of a solvation layer on the surface of the silica particles through hydrogen bonding, where the solvation layer is composed of a strongly bound inner layer and a loosely bound outer layer. **b** Sets of pictures and photographs of the dispersions with the particle volume fraction smaller than the threshold for crystallization (the left), intermediate between the thresholds for crystallization and liquid-to-solid transition (the middle), and larger than the threshold for the transition (the right). **c** Procedure for developing the photonic films by gently sandwiching the dispersions with a pair of glass slides and photocuring them. **d** Photographs of the photonic films produced from the dispersions with volume fractions of 30% (top), 40% (middle), and 50% (bottom). **e** Reflectance spectra of the photonic films before and after photocuring for volume fractions of 30%, 40%, and 50%. **f** Central wavelength at the reflectance peak, *λ*_max_, before and after photocuring (red and yellow squares, left y-axis) and normalized fwhm with the central wavelength, *Δλ/λ*_max_, after photocuring (blue squares, right y-axis) as a function of the particle volume fraction. The red solid curve is Bragg’s equation, and the yellow curve is the modified Bragg’s equation obtained through the multiplication with a factor of *α* = 0.94

For the creation of orderly colloidal particle arrays, we utilize photocurable dispersions of monodisperse silica particles in ethoxylated trimethylolpropane triacrylate (ETPTA). The silica particles with a diameter in the range of 170–220 nm spontaneously crystallize in ETPTA for a volume fraction higher than *ϕ*_th_ ~ 15% due to interparticle repulsion; this value is far below the threshold for hard spheres of 49.4%^25,37^. The repulsion is caused by a solvation layer on the surface formed by hydrogen bonds between the silanol groups on the silica and acrylate groups of ETPTA. It has been recently reported that the dispersions turn into a nonflowing solid containing glassy arrays of particles for a volume fraction higher than *ϕ*_tr_ ~ 53%, which is below the random dense packing of 64%, due to a lack of unbound ETPTA molecules^25,37^. The spontaneous crystallization and early liquid-to-solid transition indicate that the solvation layer has two distinct regions of a strongly bound inner layer and a loosely bound outer layer, as illustrated in Fig. 1a. For *ϕ* < *ϕ*_th_, no overlap among the solvation layers is expected, resulting in a disordered array of particles, as illustrated in the first panel of Fig. 1b. When *ϕ*_th_ < *ϕ* < *ϕ*_tr_, overlap of the outer solvation layer leads to the spontaneous formation of a structured array of particles characterized by long-range order, as shown in the second panel. For *ϕ* = *ϕ*_tr_, all ETPTA molecules are bound on the surface and no free ETPTA molecules are available in the interstitial regions, causing a transition from a viscoelastic liquid to a brittle solid; here, a lack of particle mobility results in the formation of glassy arrays, as shown in the third panel. The thickness of the entire layer is estimated to be approximately 59.1 nm, where the inner layer is 6.3 nm thick from the values of *ϕ*_th_ and *ϕ*_tr_^25^.

To produce photonic films and patterns, the volume fraction of the silica particles in ETPTA need to be larger than *ϕ*_th_ for spontaneous crystallization and smaller than *ϕ*_tr_ for processing. The volume fraction waas usually set to approximately 30% for most previous reports since relatively low viscosity provides ease of processing^10^. However, no systematic study on the influence of the volume fraction has been performed. By optimizing the silica volume fraction, the overlap of solvation layers can be improved, potentially enhancing the crystallinity of the non-close-packed arrays and optical properties. To determine the optimal volume fraction, we prepare dispersions with volume fractions of *ϕ* = 30%, 40%, and 50% for silica particles with a diameter of *d* = 204 nm. The film is simply prepared by gently sandwiching the dispersion with two pairs of glass slides to a thickness of 70 μm. The film is thermally annealed at 70°C for 15 minutes to improve the particle ordering before photopolymerization of ETPTA, as illustrated in Fig. 1c. Furthermore, the heat treatment slightly enhances the reflectivity, as shown in Fig. S1 of the Supplementary Information.

The photopolymerization of ETPTA results in a solid composite film in which non-close-packed arrays of silica particles are embedded in a polymerized ETPTA matrix. The films with volume fractions of 30%, 40%, and 50% exhibit red, green, and blue reflection colors for normal incident light, respectively, as shown in Fig. 1d. The change in color based on volume fraction is also supported by examining their reflectance spectra (Fig. 1e). As the volume fraction increases, the interparticle separation decreases, which causes a blueshift of the structural color. The wavelengths for the structural resonance are characterized by the reflectance spectra, as shown in Fig. 1e. The films in the liquid state before photopolymerization show reflectance peaks at 658 nm, 593 nm, and 558 nm for volume fractions of 30%, 40%, and 50%, respectively. The peak positions align well with the anticipated stopband position, *λ*_max_, derived from Bragg diffraction occurring from the (111) planes of a non-close-packed face-centered cubic (fcc) lattice, as shown in Fig. 1f:

1 $${{\rm{\lambda }}}_{\max }=2{d}_{111}{n}_{{eff}}={\left(\frac{\pi }{3\sqrt{2}\phi }\right)}^{1/3}{\left(\frac{8}{3}\right)}^{1/2}d{n}_{{eff}},$$

where d_111_ represents the distance between adjacent (111) planes and n_eff_ stands for the effective refractive index. To determine the value of n_eff_, we utilize the Maxwell−Garnett average of refractive indices of silica particles (n_silica_ = 1.45) and a liquid ETPTA (n_EPTPA_ = 1. 471):

2 $${{n}_{{eff}}}^{2}={{n}_{p}}^{2}\phi +{{n}_{m}}^{2}\left(1-\phi \right).$$

The alignment between the peak positions of the spectra and *λ*_max_ from Eq. 1 indicates that silica particles form a non-close-packed fcc structure, with parallel (111) planes to the film surfaces. Notably, the wavelengths blueshift upon photopolymerization of ETPTA for all three volume fractions, as shown in Fig. 1e. The blueshift is caused by the photopolymerization-induced shrinkage of the film along the thickness direction with minimal shrinkage along the lateral direction due to the geometry^44^. The resonant wavelengths of the polymerized films coincide with *λ*_max_ multiplied by a factor of 0.94, as shown in Fig. 1f. At the same time, photopolymerization increases the refractive index of ETPTA such that the refractive index contrast increases, which results in the enhancement of reflectivity.

The interparticle separations in the non-close-packed fcc lattice before photopolymerization are estimated as 72 nm, 47 nm, and 29 nm from the resonant wavelengths for *ϕ* values of 30%, 40%, and 50%, respectively. Therefore, the interparticle repulsive force that the particle experiences is expected to be different. Nevertheless, no significant difference in the optical properties of the polymerized composite films for the three different volume fractions are observed. The peak heights are approximately 40%, and the full width at half-maximums (fwhm) normalized by the central wavelength of the peak, ∆*λ*/*λ*_max_, is approximately 0.025, signifying a minor difference in ordering. Although particles at higher volume fractions are expected to experience stronger repulsive forces at shorter interparticle separations, the higher viscosity of the dispersion suppresses the rearrangement of particles more, which potentially results in no meaningful difference in the structural ordering and optical properties. The viscosity of the dispersions at a low shear rate is measured to be 118 GPa·s, 147 GPa·s and 262 GPa·s for 30%, 40%, and 50%, respectively, where all three dispersions have shear-thinning behavior, as shown in Fig. S2.

### Shear-induced reorganization of particles

**Fig. 2:**
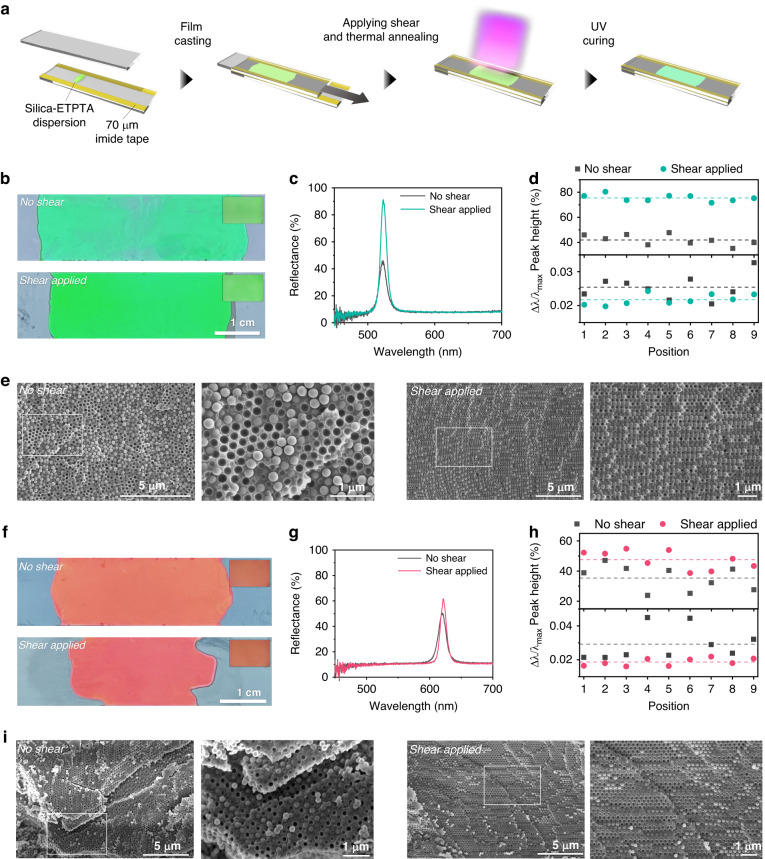
Shear-induced alignment of the colloidal crystals. **a** Procedure for making photonic films by gently sandwiching the dispersions with a pair of glass slides, shearing, and photocuring them. **b** Photographs of the photonic films prepared from the dispersion with a volume fraction of 50% without shear (top) and with shear applied at 1 s^−1^ (bottom). Insets are the optical microscopy images of the corresponding photonic films. **c** Reflectance spectra of the photonic films without and with shear. **d** Peak height from baseline (top) and normalized fwhm (bottom) at the 9 different positions of the photonic films. The horizontal dotted lines are average values. **e** SEM images showing the cross-sections of the photonic films prepared without shear (the left) and with shear (the right) taken at two different magnifications. **f–i** Same sets as (**b**–**e**) but for a volume fraction of 30%

The high viscosity at a high volume fraction restricts particle motion, which results in the formation of polycrystalline structures consisting of small domains despite strong interparticle repulsion^25^. To facilitate the rearrangement of the particles and unify the crystal orientation, the dispersions sandwiched by a pair of glass slides are sheared by moving the top slide while fixing the bottom slide, as illustrated in Fig. 2a. To control the speed, we use a syringe pump, as shown in Fig. S3. For the dispersion with *ϕ* = 50%, the shear flow drastically improves the optical performance and film uniformity, while the color and the position of the reflectance peak remain consistent, as shown in Fig. 2b. Although the colors of the film in the photographs appear slightly different due to a slight change in the imaging conditions, those in the optical microscopy images are consistent. The film produced without shear shows uneven and mottled coloration, lacking sharpness and appearing slightly dim. Conversely, the film subjected to a shear rate of 1 s^−1^ shows a brighter and more vivid color with high uniformity. This enhancement can be quantified with the reflectance spectra, as shown in Fig. 2c. For the 9 different positions on the surface, the average peak height from the baseline is increased from 42% without shear to 76% with shear, and the value of ∆*λ*/*λ*_max_ decreases from 0.025 to 0.022, as shown in Fig. 2d; the reflectance spectra measured at the 9 different positions are shown in Fig. S4a, b. Specifically, the peak transforms into a taller and sharper peak by shear flow, which occurs in all areas of the film. The coefficients of variation (CVs) are calculated by dividing the standard deviation by the mean value, which serves as an indicator of the uniformity of the film. The CVs for the peak height and ∆*λ*/*λ*_max_ are 0.038 and 0.068 for the shear-applied samples, respectively, whereas the CVs are 0.094 and 0.14 for the no shear; these results indicate that the uniformity is enhanced by the applied shear. No shift in *λ*_max_ is observed, as shown in Fig. S4c,d. The enhancement of the optical properties and uniformity originates from the improved crystallinity and unified orientation of the crystals in the shear-applied film. Without shear flow, the particles produce crystalline arrays along the top and bottom surfaces, which span approximately 20 µm, as shown in Fig. S5a; the crystalline arrays are produced by shear during the sandwiching of the dispersion with a pair of glasses. The central part of the film contains poorly ordered arrays or well-ordered arrays at very small domains whose orientations are somewhat random, as shown in the left two panels of Fig. 2e. As the thickness of the crystalline array from the top and bottom fluctuates position-by-position, the reflectivity also fluctuates. When the dispersion is properly sheared, the particles are regularly ordered over an entire thickness of 70 µm, as shown in Fig. S5b. The magnified view shows the single-crystalline structure in the right two panels of Fig. 2e. As the film contains a unidirectional orientation of colloidal crystals across the entire cross-section regardless of position, the reflectivity is much higher, and its fluctuation is much lower. The crystalline arrangement produced by shear and thermal annealing is highly stable without photocuring as long as the dispersion is not mechanically perturbed. We confirm this by incubating the shear-applied dispersion in a light-blocked state and curing it after several days. The photonic film displays a high reflectivity consistent with immediate curing, as shown in Fig. S6.

We investigate the influence of the shear flow for the dispersion with *ϕ* = 30%, as shown in Fig. 2f. Although the reflectivity increases and ∆*λ*/*λ*_max_ decreases with shear, the enhancement is incremental, as shown in Fig. 2g. Furthermore, film uniformity is relatively poor in comparison with *ϕ* = 50% even after applying the shear flow, as shown in Fig. 2h; the reflectance spectra measured at the 9 different positions are shown in Fig. S8a,b. The CVs for the peak height and ∆*λ*/*λ*_max_ are 0.15 and 0.13, respectively, for the shear-applied samples, which are larger than those for the samples with *ϕ* = 50%. Shear aids in achieving a more regular particle arrangement even at low volume fractions, as shown in Fig. 2i. However, this effect is more pronounced at higher volume fractions. As we discussed, the lack of particle mobility at high volume fractions restricts the rearrangement of particles. The shear flow facilitates the positioning of the particles in energetically favorable crystalline arrays under strong interparticle repulsion. In contrast, particles have relatively high mobility for rearrangement at low volume fractions, but weak repulsion results in less ordered arrays. Therefore, the shear flow is unable to significantly improve the crystallinity.

The presence of a solvation layer around the silica particles, which generates interparticle repulsion, helps maintain the dispersion stability of inks for all three different volume fractions of 30%, 40%, and 50%. Even after one year of storage, the inks show no phase separation or aggregation, as shown in Fig. S7a. The high stability is further confirmed by reflectance spectra of the photonic films made using one-year-old inks; these results are comparable with those made using as-prepared inks, as shown in Fig. S7b. This high ink stability originates from solvation layer-induced interparticle repulsion.

**Fig. 3:**
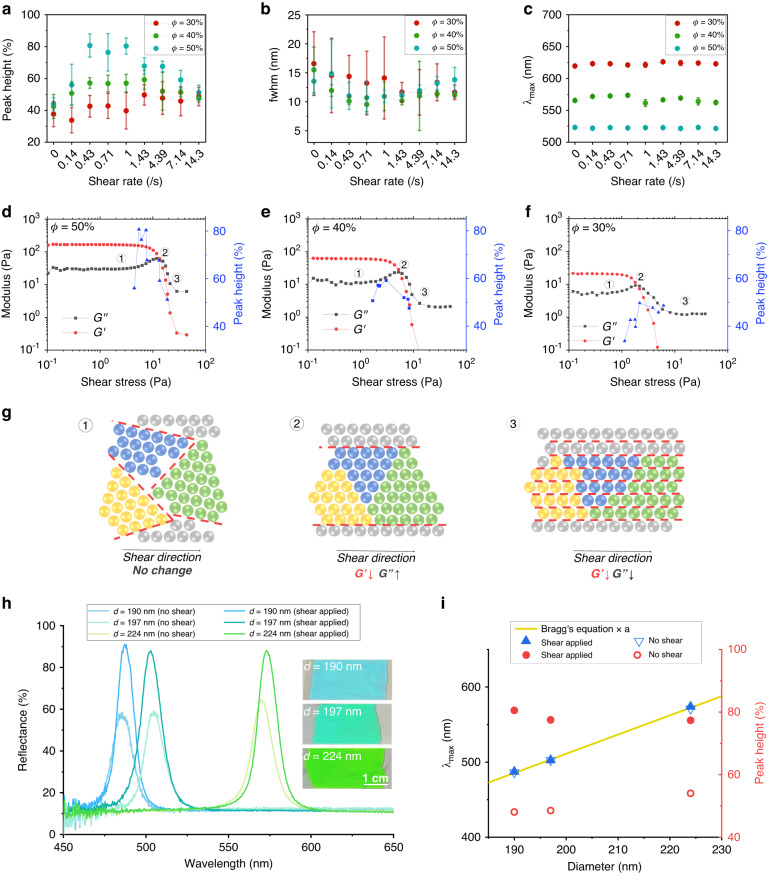
Optimum shear conditions. **a–c** Reflectance peak height from the baseline, fwhm, and peak wavelength for the volume fractions of 30%, 40%, and 50% as a function of the shear rate. **d–f** Storage modulus (G’) and loss modulus (G”) of the dispersions with volume fractions of 50%, 40%, and 30% (the left y-axis) and peak height (right y-axis) as a function of the shear stress. **g** Pictures showing the particle arrangement expected in the three distinct regions of the linear viscoelastic region, loss-modulus overshoot, and the reduction of both moduli. **h** Reflectance spectra of the photonic films prepared from the dispersions of the silica particles with diameters of 190, 197, and 224 nm at a volume fraction of 50% with and without shear. Insets are photographs of the corresponding photonic films prepared with shear. **i** Central wavelength at the reflectance peak, *λ*_max_, (unfilled inverted triangles and filled triangles, left *y*-axis) and peak height (unfilled circles and filled circles, right *y*-axis) of the photonic films prepared with and without shear at 1 s^−1^ as a function of the particle diameter. The yellow curve represents the modified Bragg’s equation with a multiplication factor *α* of 0.94

To systematically study the influence of the shear flow and optimize the shear rate, we apply a wide range of shear rates from 0.14 s^−1^ to 14.3 s^−1^ on the dispersions with *ϕ* = 30%, 40%, and 50% and characterize the optical properties of the resulting films, as shown in Fig. 3a, b. Notably, higher volume fractions exhibit more pronounced enhancements in the optical performance owing to the rearrangement induced by shear, whereas lower volume fractions show relatively minor variations. At *ϕ* = 50%, the peak height from the baseline increases from 44% to 80.7% as the shear rate increases from 0 to 0.43 s^−1^ and stays at approximately 80% up to a shear rate of 1 s^−1^. For higher shear rates, the peak height gradually decreases to 51.2%. The fwhm has the opposite trend and is relatively small at shear rate range of 0.43 s^−1^ to 1 s^−1^, where the peak height is maximized. At *ϕ* = 40%, the maximum enhancement of peak height by shear is approximately 17% in the shear rate range of 0.43 s^−1^ to 1.43 s^−1^, where the value of fwhm is minimized. The maximum enhancement of the peak height is as low as 12%, which occurs at shear rate range of 1.43 s^−1^ to 14.3 s^−1^ at *ϕ* = 30%. Specifically, the shear-induced enhancement of the optical performance is maximized at a 50% volume fraction, where the optimum shear rate range is 0.43 s^−1^ to 1 s^−1^. Notably, the peak position remains exclusively governed by the volume fraction and remains unaffected by the shear influence, as shown in Fig. 3c.

To comprehensively investigate the influence of the shear flow on particle rearrangement, we conduct a comparative analysis of the rheological properties across three different volume fractions, as shown in Fig. 3d–f. For all three volume fractions, the storage modulus (G’) and the loss modulus (G”) change along with shear stress in a similar manner. A linear viscoelastic region with nearly constant G’ and G” for low shear stress is present, indicating minimal change in the colloidal arrangement. In this linear region, the dispersion is expected to consist of small crystallites with varying orientations. As shear stress increases, a loss-modulus overshoot is observed due to the build-up of crystalline structure caused by shear-induced alignment of the small crystallites along the flow direction. This alignment leads to fusion with neighboring crystallites, reducing the grain boundaries and increasing the loss modulus by concentrating the shear flow at local regions. A decrease in both G’ and G” is followed for higher shear stress and indicates the breakdown of the crystalline domains into a sheet-like arrangement^46–50^. The particle arrangement in the three distinct regions is schematically illustrated in Fig. 3g. Although the three regions are commonly observed for all cases, the moduli notably increase and the linear viscoelastic region is extended up to a larger shear stress for a higher volume fraction.

To determine the correlation between the rheological response and the optical performance, the peak height is incorporated into the modulus graph by converting the shear rate into shear stress, as shown in Fig. 3d–f; the conversion is based on the shear stress as a function of the shear rate in the continuous rotation mode from Fig. S9. For *ϕ* = 50%, a significant enhancement of the peak height is observed at the transition from the linear viscous region to the loss-modulus overshoot region, and the peak height drastically decreases near the overshoot, as shown in Fig. 3d. For relatively low shear stress, the overall structures composed of small crystallites with diverse orientations remain unchanged such that no change in the moduli occur. As the shear stress further increases, a gradual increase in the loss modulus and a gradual decrease in the storage modulus are observed as the small crystallites begin to undergo reorganization along with the flow direction. The loss modulus reaches a maximum when the crystallites are fused by reducing the number of grain boundaries. The particles in the highly fused crystallites, corresponding to the loss-modulus overshoot, have relatively low mobility, as the particle-free voids are concentrated along the reduced boundaries. Therefore, no significant enhancement of arrangement occurs by subsequent thermal annealing. In contrast, the particles have sufficient mobility and shear effects at the onset of the loss-modulus increase where the small crystallites are weakly fused. Therefore, thermal annealing of the sheared dispersions facilitates the growth of crystallites into larger crystalline domains with unidirectional alignment, thereby significantly enhancing the optical properties. When the dispersion is photocured without thermal annealing immediately after optimal shearing, the film exhibits a reflectance peak height of approximately 65%, as shown in Fig. S10. Specifically, thermal annealing enhances the peak height by 10% under the optimum shear condition. Therefore, the optimum shear condition occurs before the overshoot in the loss modulus. This trend is observed for *ϕ* = 40% even though the peak height is generally lower, as shown in Fig. 3e. In contrast, the peak height gradually increases up to the shear condition that occurs with the loss-modulus overshoot for *ϕ* = 30%, as shown in Fig. 3f. Since the particle mobility is high enough for rearrangement even at the overshoot and the interparticle repulsion for crystallization is relatively weak for *ϕ* = 30%, the shear-induced growth of the crystalline domain is more advantageous than the thermal annealing-induced growth.

The optimum shear rate for the 50% volume fraction is valid not only for the particles with an average diameter of 204 nm but also for other sizes. For example, we achieve significant enhancement for particle diameters of 190, 197, and 224 nm at a volume fraction of 50% with a shear rate of 1 s^−1^, as shown in Fig. 3h. Consistent with previous results, the films created under shear display narrower reflection peaks with increased height. Additionally, the films produced with shear exhibit vivid and uniform colors. The reflectance peak positions follow Eq. (1) multiplied by a factor of 0.94, as shown in Fig. 3i. The peak height from the baseline increases from approximately 50% to 80% with the shear. Shear-assisted crystallization can be utilized not only for films with a thickness of 70 µm but also for thinner or thicker films, as illustrated in Figs. S11a, b. The shear rate is set to 1 s^−1^ for all thicknesses by adjusting the speed of the top slide. The reflectivity decreases when the film becomes thinner than 70 µm, and no substantial enhancement is observed for the thicker film. Therefore, Bragg’s attenuation length is expected to be approximately 70 µm. This long attenuation length is attributed to the small refractive index contrast between the silica particles and polymerized ETPTA, which is approximately 0.02. The films show a blueshift of the stopband along with an angle for specular reflection, as shown in Fig. S11c, d. During the blueshift, the reflectivity remains high up to an angle of 40° and decreases for higher angles.

### High-quality structural color patterns and color mixing

**Fig. 4:**
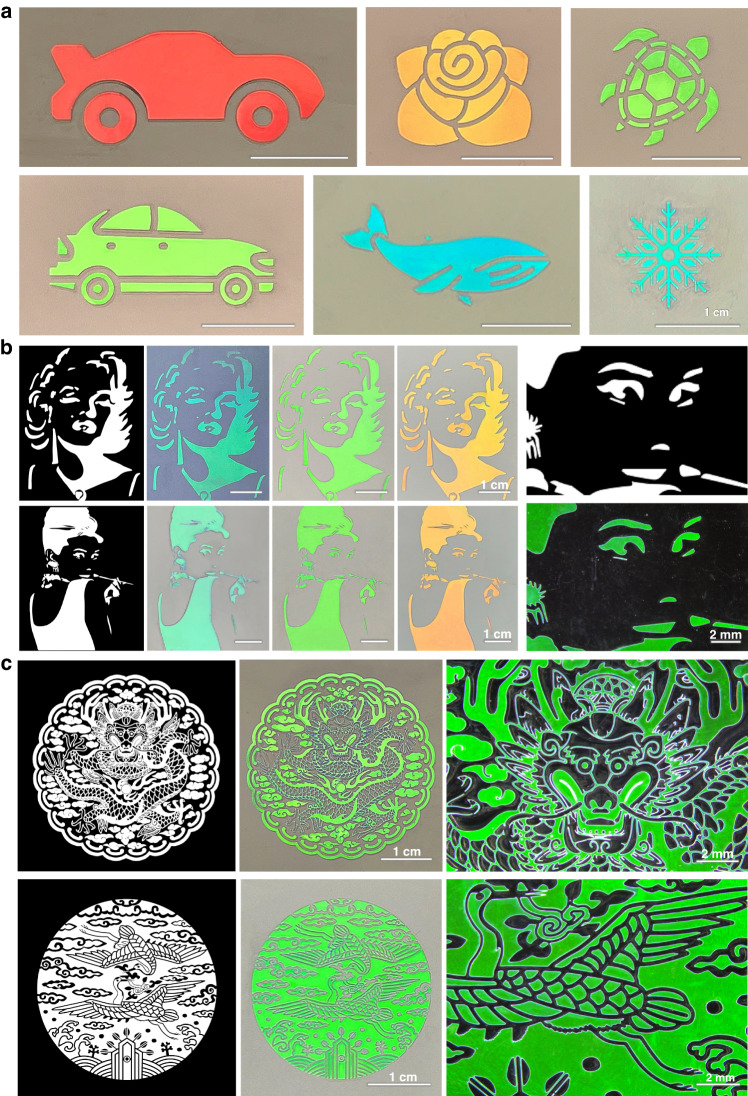
Micropatterning of the photonic crystals. **a** Photographs of the structurally colored graphics: red car, orange rose, green turtle, green car, cyan whale, and cyan snowflake. **b** Sets of the photomask design and photographs of the corresponding photonic patterns with the three different colors for the graphics of Marylin Monroe (top left) and Audrey Hepburn (bottom left). The photomask design and resulting pattern for the face of Audrey Hepburn are consistent (right). **c** Sets of the photomask design and photographs of Korean traditional patterns of a dragon in embossing (top) and a pair of cranes in intaglio (bottom). The magnified views show detailed features of the patterns (right)

The high crystallinity of silica particles in entire areas under optimum conditions produces high optical performance and uniformity. With the optimum conditions, we created various color patterns using photolithography^10,14,18,26,37^. Since the dispersions are photocurable, regioselective UV irradiation through a photomask followed by washing of the uncured dispersion results in the structural color patterns. These patterns have the same graphics as the transparent regions of the photomask. To achieve this, we placed a photomask on the top glass slide. After applying shear, we exposed the film of the dispersions to UV light through these photomasks. We then removed the photomasks and top glass slide from the bottom glass plate and washed away the uncured dispersions. The resulting photonic patterns remained on the glass plate, displaying vivid structural colors determined by the diameter of silica particles. For instance, we created designs such as a red car, orange rose, green turtle, green car, cyan whale, and cyan snowflake, as shown in Fig. 4a.

More intricate patterns are also possible. For instance, we create a graphic of Marilyn Monroe and a graphic of Audrey Hepburn in three colors: cyan, green, and orange, as depicted in Fig. 4b. The intricate features of the photomasks effectively transfer to the photonic patterns, as evident in magnified images. We observe minimal hue and brightness variation across the entire color pattern area because we utilize a dispersion with *ϕ* = 50%, where a shear rate of 1 s^−1^ is used. Larger, more complex patterns are also achieved through photolithography, maintaining high optical performance and color uniformity. Examples include Korean traditional patterns of a dragon in an embossing and a pair of cranes in intaglio, as shown in Fig. 4c. The minimum feature size of the micropatterns is estimated from approximately 25 µm from the radius of the rounded corners in the acute patterns and the reduction in the amplitude of wavy patterns, as shown in Fig. S12. The resolution can be improved by integrating the photomask on the top slide, which provides close contact between the photomask and dispersion and minimizes the scattering at the edges. These high-quality patterns using dispersions with *ϕ* ~ 30% or other methods are difficult to produce.

**Fig. 5:**
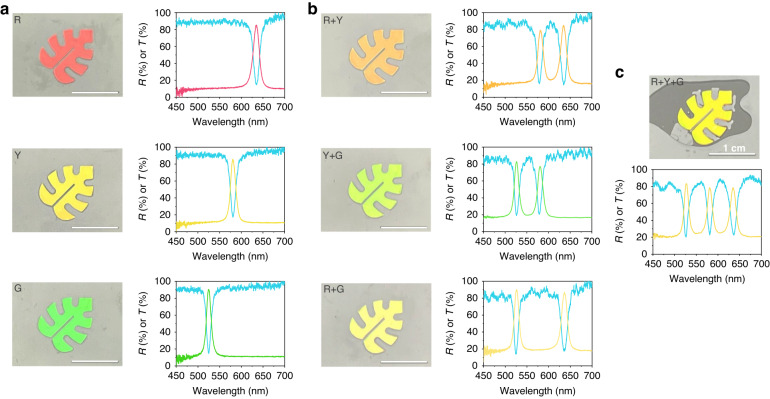
Multilayered photonic patterns. **a** Sets of photographs and reflectance and transmittance spectra of single-layered photonic leaf patterns with red (top), yellow (middle), and green (bottom) colors. **b** Same set as (**a**) but for double-layered photonic leaf patterns with a combination of red and yellow (top), yellow and green (middle), and green and red (bottom). **c** Same set as (**a**) but for a triple-layered leaf pattern

Films and patterns exhibit high reflectivity at the stopband position due to the high crystallinity of the silica particles. Simultaneously, they are highly transparent outside of the stopband because the unidirectionally ordered crystals with a small refractive index contrast between polymerized ETPTA and silica particles reduce the diffusive scattering of light. We produce red, yellow, and green leaf patterns using the dispersions with *d* = 240 nm at *ϕ* = 45%, *d* = 228 nm at *ϕ* = 50%, and *d* = 204 nm at *ϕ* = 50%, respectively. These patterns show uniform colors with a single reflectance peak at 635 nm, 581 nm, and 524 nm, as shown in Fig. 5a. The reflectivity at the stopband reaches approximately 85% for all three patterns. The transmittance spectra show a dip in the stopband, with a low transmittance of 15%. The transmittance increases to approximately 90% for wavelengths shorter than the stopband and even surpasses 90% for longer wavelengths. Achieving this high transmittance, especially for shorter wavelengths, is challenging with *ϕ* ~ 30%^10^. The sum of reflectance and transmittance is close to 100%, indicating negligible diffusive scattering, as shown in Fig. S13.

This high transparency enables the films or patterns to be stacked; this develops multiple reflectance peaks without a significant reduction in the reflection intensity. For example, we stack double layers of two different leaf patterns, resulting in two distinct reflection peaks and mixed structural colors, as depicted in Fig. 5b. The double layer of red and yellow patterns displays an orange color with two pronounced peaks at 635 and 581 nm. Similarly, combining yellow and green produces a mint pattern with two peaks at 581 and 524 nm, while mixing red and green provides a banana color with two peaks at 635 and 524 nm. All combinations show two dips in their transmittance spectra at the wavelengths for the reflectance peaks, with no noticeable changes in dip depth. Combining all three colors of red, yellow, and green results in bright yellow, as shown in Fig. 5c. As anticipated from the high transparency, the three distinct peaks and dips appear in the reflectance and transmittance spectra, respectively. Notably, measuring the color and reflectance spectrum from both sides of the double and triple layers shows negligible differences, as shown in Fig. S14. This consistency reaffirms the high transparency of each layer except at the stopband wavelengths. The multiple peaks and dips in the stacked films or patterns hold promise as invisible optical barcodes for anticounterfeiting purposes. Since the peak position changes with the diameter of the silica particles and the number of stacking layers is not limited to 3 but can further increase, numerous optical barcodes can be generated^10,40,41^.

## Conclusion

In this study, we examine the effect of the volume fraction of silica particles in photocurable dispersions and the role of the shear flow on the optical performance and uniformity of the photonic films and patterns. Using a particle volume fraction close to 30%, which is a standard in conventional methods due to its low viscosity, leads to modest reflectivity and acceptable fluctuations in the reflectivity. Introducing shear flow incrementally enhances this outcome. In contrast, when the volume fraction approaches 50% combined with the optimized shear conditions, the results show high reflectivity and uniformity. Reflection peaks increase to approximately 80% with respect to the baseline, with a minimal CV value of 0.038. Impressively, the transmittance reaches up to 90%, even at off-resonant wavelengths shorter than the stopband. This high reflectivity at the stopband, paired with the transparency at off-stopband wavelengths, stems from the high crystallinity and uniform orientation. These factors increase structural resonance and reduce diffusive scattering. At elevated volume fractions, the particles experience considerable repulsive forces but exhibit limited mobility due to the increased viscosity, leading to the polycrystalline structures. The introduced shear flow aids the particles in aligning into unidirectionally ordered crystals along the flow direction. Thus, the shear-induced unification of the crystal orientation, especially at ultrahigh volume fractions, results in superior optical performance, consistency, and reproducibility. This advancement facilitates more sophisticated applications. Additionally, colloidal photonic crystals can be micropatterned, facilitating the creation of expansive and intricate photonic graphics with remarkable uniformity using photolithographic techniques. The high transparency of these structures supports the stacking of the photonic films and patterns, enabling multilayered designs. These photonic crystals show mixed structural colors, multiple reflectance peaks, and multiple transmittance dips without sacrificing intensity. These patternable and stackable photonic crystals with high optical performance and uniformity hold significant promise for a broad spectrum of optical applications, ranging from optical filters and reflectors to optical barcodes. For example, multiple anticounterfeiting optical barcodes are promising applications for these photonic crystals. Their enhanced wavelength selectivity enables the development of intricate and secure patterns, vital for counterfeit prevention and secure identification. Additionally, using these crystals enhances the optical filter and reflector performance, providing innovative solutions across industries requiring precise spectral control.

## Materials and methods

### Preparation of dispersions

Monodisperse silica particles with average diameters of 190, 197, 204, 224 nm, 228 nm, and 240 nm (Sukgyung AT) were washed with ethanol several times and then dried to measure the weight. The silica powders were redispersed in ethanol (99.5%, Merck) using sonication overnight, and the ethanolic dispersions were combined with ethoxylated trimethylolpropane triacrylate (ETPTA, Mn ~428 g mol^−1^, Sigma‒Aldrich) containing 1 w/w% photoinitiator (Darocur 1173, Sigma‒Aldrich). The amounts of ETPTA were adjusted to achieve volume fractions of silica particles at 0.3, 0.4, and 0.5 in the ethanol-free base, and the densities of silica particles and ETPTA were 2.0 and 1.11 g/cm^3,^ respectively. The mixture was sonicated for 10 min and then kept in a convection oven at 70 °C overnight to completely evaporate the ethanol. The resulting dispersions were defoamed and mixed using a planetary centrifugal mixer (AR-310, Thinky).

### Film casting and micropatterning

The silica-ETPTA dispersion was placed on a glass slide with two 70-μm-thick spacers of polyimide tape (Kapton) arranged parallel to the longer side of the glass slide. Subsequently, the dispersion was carefully covered with another glass slide, ensuring even spreading of the dispersion. To apply a constant shear rate, the bottom glass slide was fixed, and the top glass slide was pulled at a consistent rate using a syringe pump (Legato 100, KDS), where an alumina plate (LabCeramic) with a mass of 98 g was placed on the top slide to prevent the separation of two glass slides. The pulling rate was controlled in a wide range with the syringe pump. After applying the shear, the glass slide was secured to prevent any further movement and then heated in a 70 °C oven for 15 min. Finally, UV light with an intensity of 21 mW/cm^2^ was irradiated on the slide for 4 s to polymerize ETPTA. For micropatterning, the bottom glass slide was pretreated with 3-(trimethoxysilyl)propyl methacrylate (Sigma‒Aldrich) to enhance adhesion of the resulting pattern structures on the glass slide. A photomask was placed on the top glass slide, through which the UV light was irradiated. After removing the photomask and the top glass slide, any unpolymerized dispersion was washed away using ethanol.

### Analysis of rheological properties

The rheological properties of the silica-ETPTA dispersion were analyzed using a rheometer (MCR 101, Anton Parr). A plate-to-plate geometry was used for dispersions with a volume fraction of silica particles of 0.40 and 0.50, whereas a Couette geometry was used for dispersions with a lower fraction of 0.30. Oscillatory tests for measuring the storage and loss moduli were carried out in stress-controlled mode. For the stress-controlled mode, the shear stress was varied in the range of 0.001 to 100 Pa through an amplitude sweep, and the frequency was fixed at 1 Hz. The viscosity was measured for shear rates spanning 0.0001 to 50 s^−1^. Before each measurement, to erase any history, the dispersions were sheared at a rate of 1 s^−1^ for 1 min and then left shear free for 2 min.

### Characterization

The photonic crystal films and patterns were imaged using consumer-grade digital cameras and observed through optical microscopy in reflection mode (Eclipse L150, Nikon) with a stereo microscope (SMZ745T, Nikon). Reflectance spectra were recorded using a fiber-coupled spectrometer (USB 4000, Ocean Optics) installed in a microscope with a 10× lens; here, a broadband dielectric mirror (BB3-E02, Thorlabs) was used as a reference for the measurements. The arrangement of silica particles in the photocured films was observed using scanning electron microscopy (SEM; S-4800, Hitachi) after the specimens were coated with osmium.

### Supplementary information

Supplemental Material

## Data Availability

The data supporting the findings of this study are accessible upon reasonable request from the corresponding author.
