# Supplementary material for: Hyperreflective photonic crystals created by shearing colloidal dispersions at ultrahigh volume fraction
Source: Microsyst Nanoeng. 2024 Jan 31;10:21. doi: 10.1038/s41378-024-00651-2 (PMC10827709; doi:10.1038/s41378-024-00651-2)
Supplement: Supplementary file 1 — Supplemental Material [file 41378_2024_651_MOESM1_ESM.docx]

**Supplementary Information**

**Hyperreflective Photonic Crystals created by Shearing Colloidal Dispersions at Ultrahigh Volume Fraction**

Minji Kim, Jong Bin Kim, and Shin-Hyun Kim*

Department of Chemical and Biomolecular Engineering, Korea Advanced Institute of Science and Technology (KAIST), Daejeon 34141, Republic of Korea

*e-mail: [kim.sh@kaist.ac.kr](mailto:kim.sh@kaist.ac.kr)

**This file includes:**

Supplementary Figures 1 to 8

**Hyperreflective Photonic Crystals created by Shearing Colloidal Dispersions at Ultrahigh Volume Fraction**

Minji Kim, Jong Bin Kim and Shin-Hyun Kim

**Supplementary Figures**

**Fig. S1. Effect of heat treatment.** Reflectance spectra of the photonic films before and after annealing at 70°C for 15 minutes for the volume fractions of 30%, 40%, and 50%.

**Fig. S2. Shear-thinning behavior.** Viscosity of dispersions with volume fractions of 30%, 40%, and 50% for silica particles with a diameter of *d* = 204 nm as a function of shear rate.


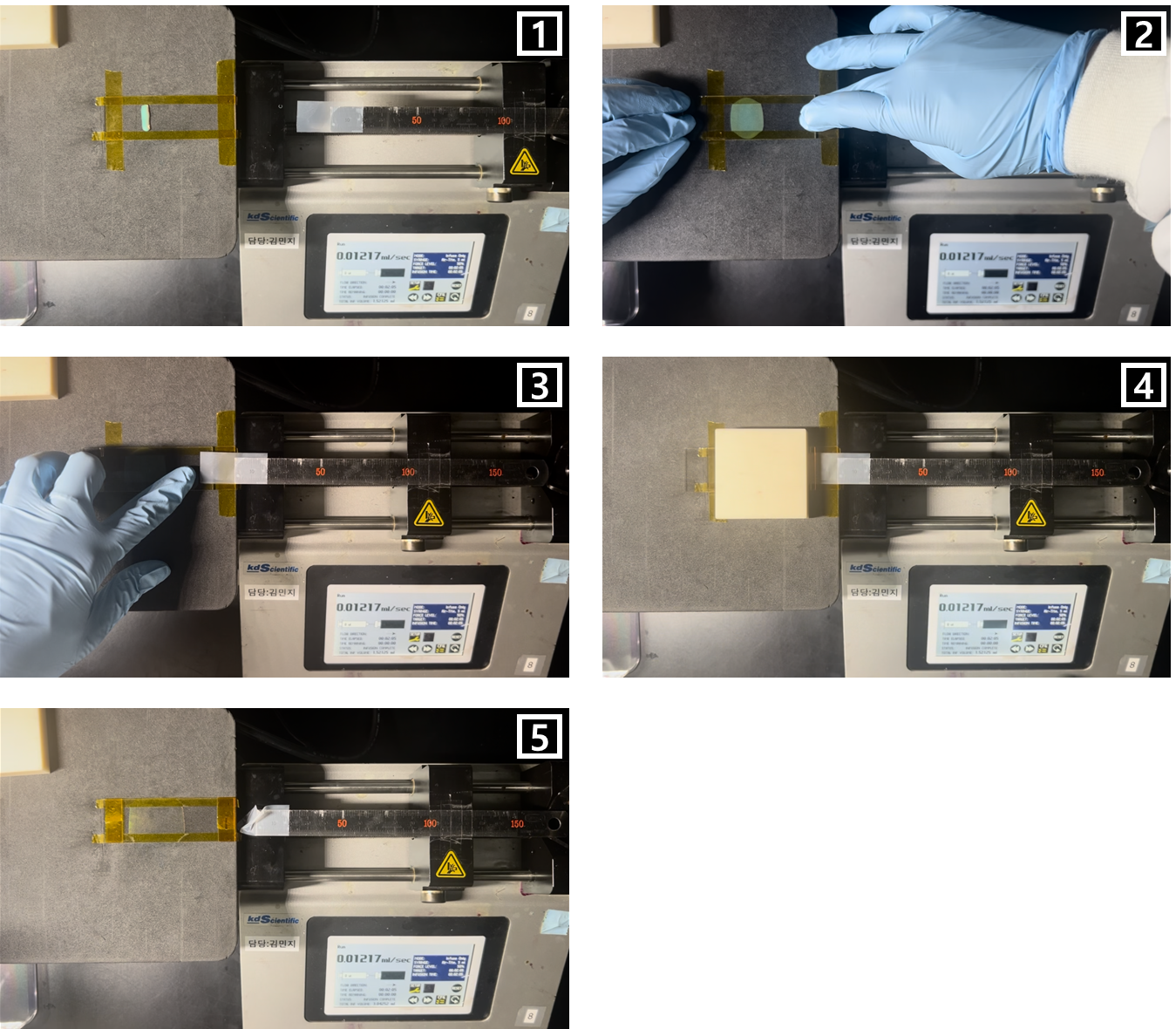


**Fig. S3. Procedure of applying shear.** Place silica-ETPTA dispersions on a glass slide with two 70-μm-thick spacers of polyimide tape arranged parallel to the longer side of the glass slide (1). Cover the dispersions with another glass slide, ensuring even spreading (2). Place an alumina plate on the top slide to prevent the separation of two glass slides (3). Pull the top glass slide at a consistent rate using a syringe pump while keeping the bottom glass slide fixed (4). Remove the alumina plate and secure the glass slide to prevent any further movement (5).


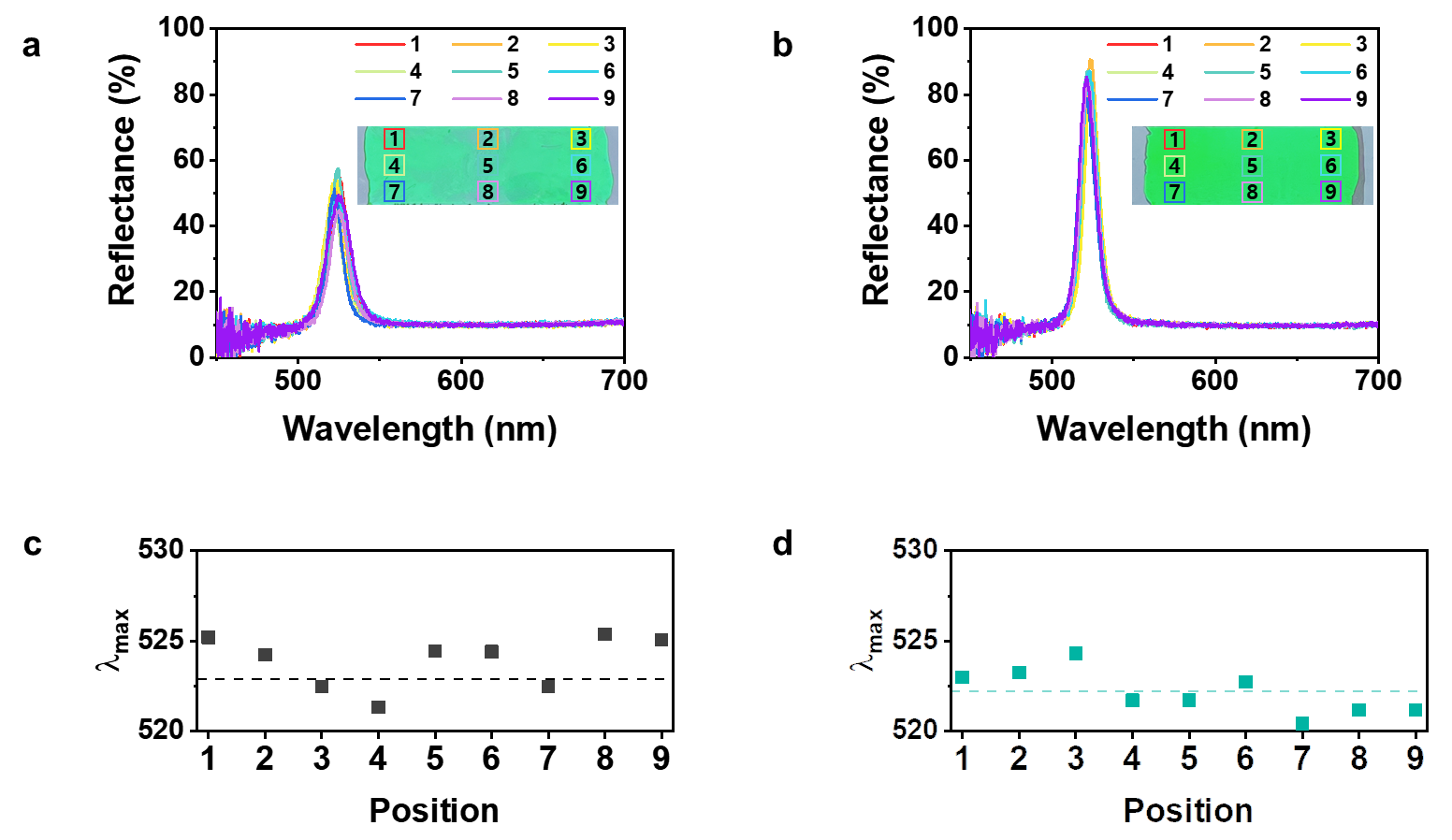


**Fig. S4. Uniformity of photonic film with volume fraction of *ϕ* = 50%. a-b** Reflectance spectra of photonic films without shear (a) and with shear applied at 1 /s (b) measured at 9 different positions. Insets are photographs of the 9 different positions in each corresponding photonic film. **c-d** *λ*_max_ at 9 different positions of the photonic films without shear (c) and with shear (d). The horizontal dotted lines are average values.


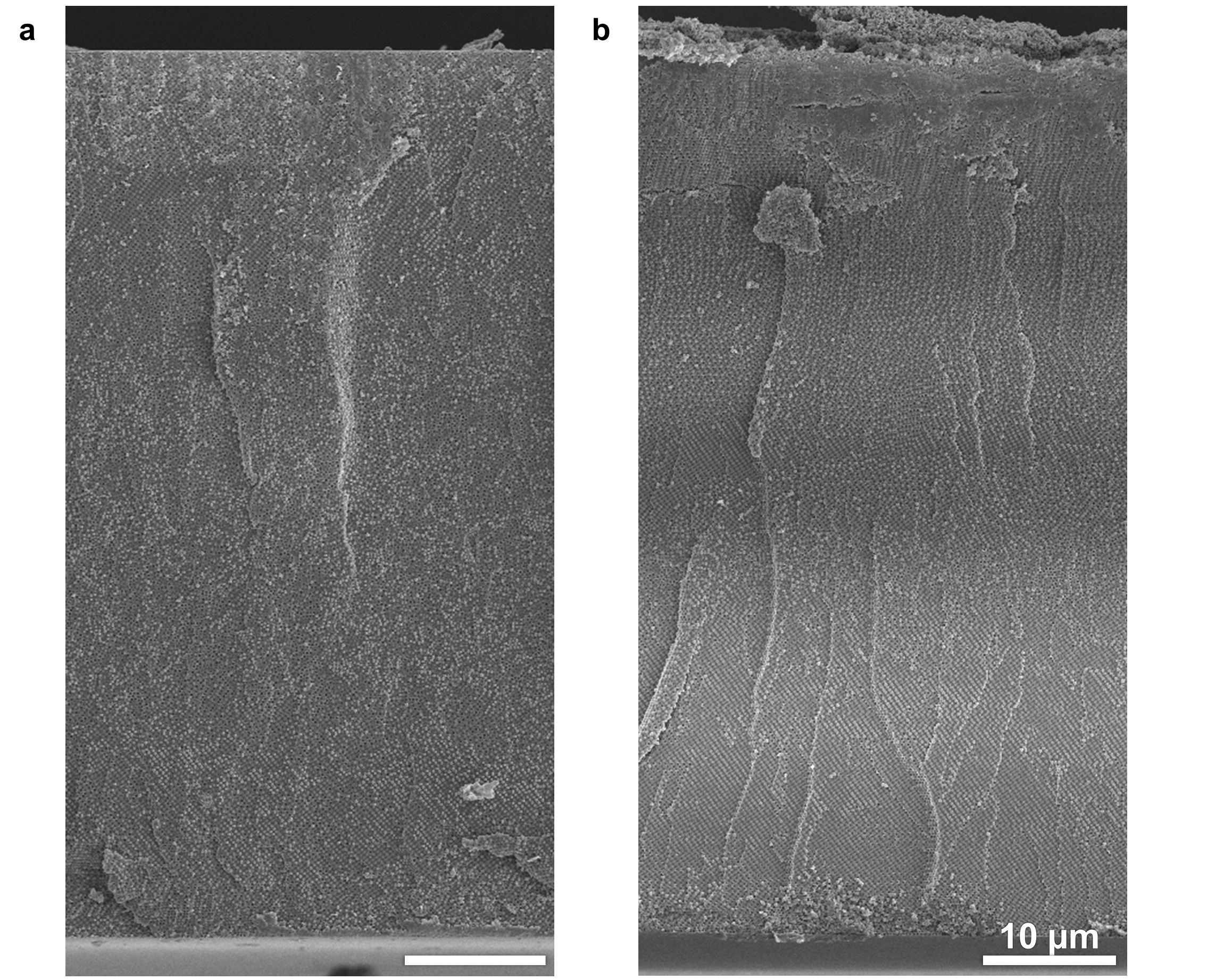


**Fig. S5. Cross-section of photonic film with volume fraction of *ϕ* = 50%. a-b** SEM images showing the cross-sections of photonic films prepared without shear (the left) and with shear (the right) taken at two different magnifications.

**Fig. S6. Stability of crystalline arrays in uncured resin.** Reflectance spectra of photonic films cured immediately following shear application and thermal annealing and after 5 days of incubation.


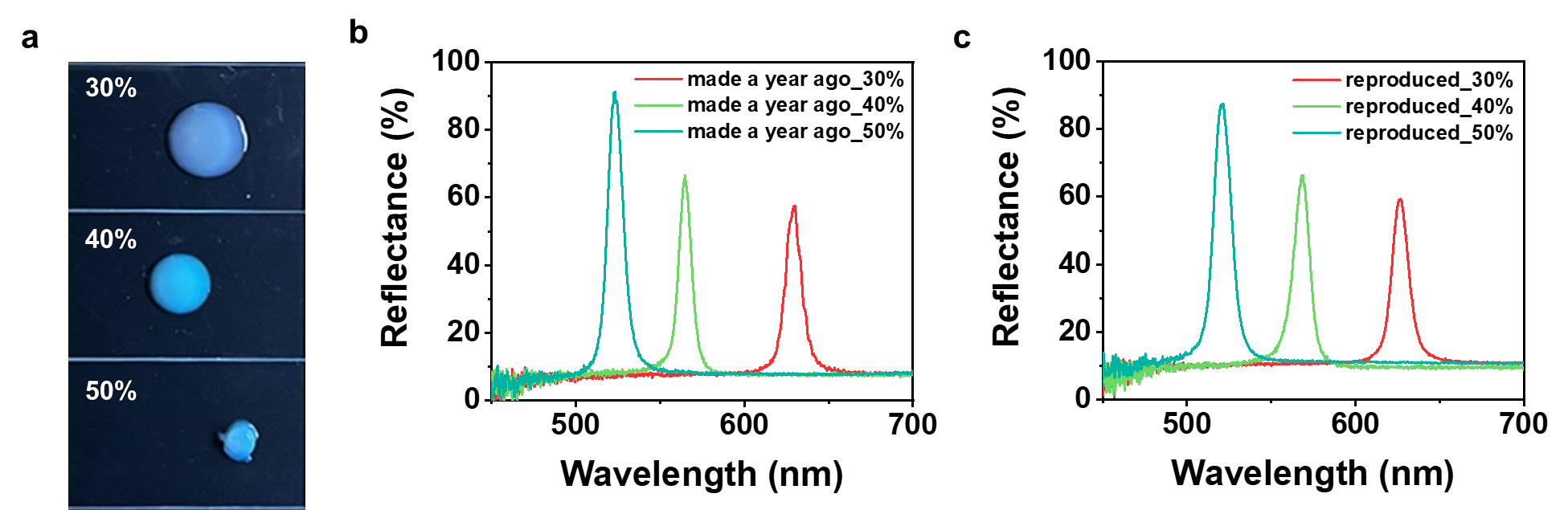


**Fig. S7. Stability of silica-ETPTA dispersions. a** Photographs of silica-ETPTA dispersions at volume fractions of 30% (top), 40% (middle), and 50% (bottom), prepared one year ago. **b** Reflectance spectra of shear-applied photonic films made and measured one year ago. **c** Reflectance spectra of shear-applied photonic films reproduced from photonic ink prepared a year ago.


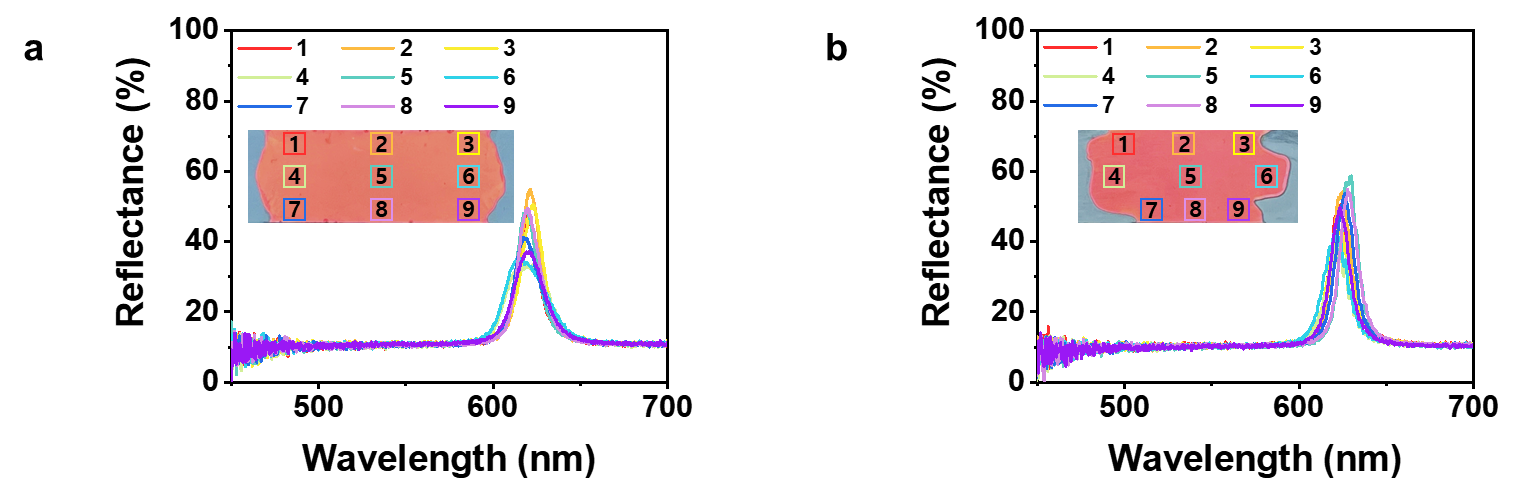


**Fig. S8. Uniformity of photonic film with volume fraction of *ϕ* = 30%. a-b** Reflectance spectra of photonic films without shear (a) and with shear applied at 1 /s (b) measured at 9 different positions. Insets are photographs of the 9 different positions in each corresponding photonic film.

**Fig. S9. Conversion of shear rate to shear stress.** Shear stress of dispersions with volume fractions of 30%, 40%, and 50% as a function of shear rate. The conversion of shear rate to shear stress is based on the relationship between shear stress and shear rate measured in continuous rotation mode.

**Fig. S10. Effect of thermal annealing at optimum shear condition.** Reflectance spectra of the photonic film prepared from the optimally shear-applied dispersion with the volume fraction of 50% without and with thermal annealing.

**
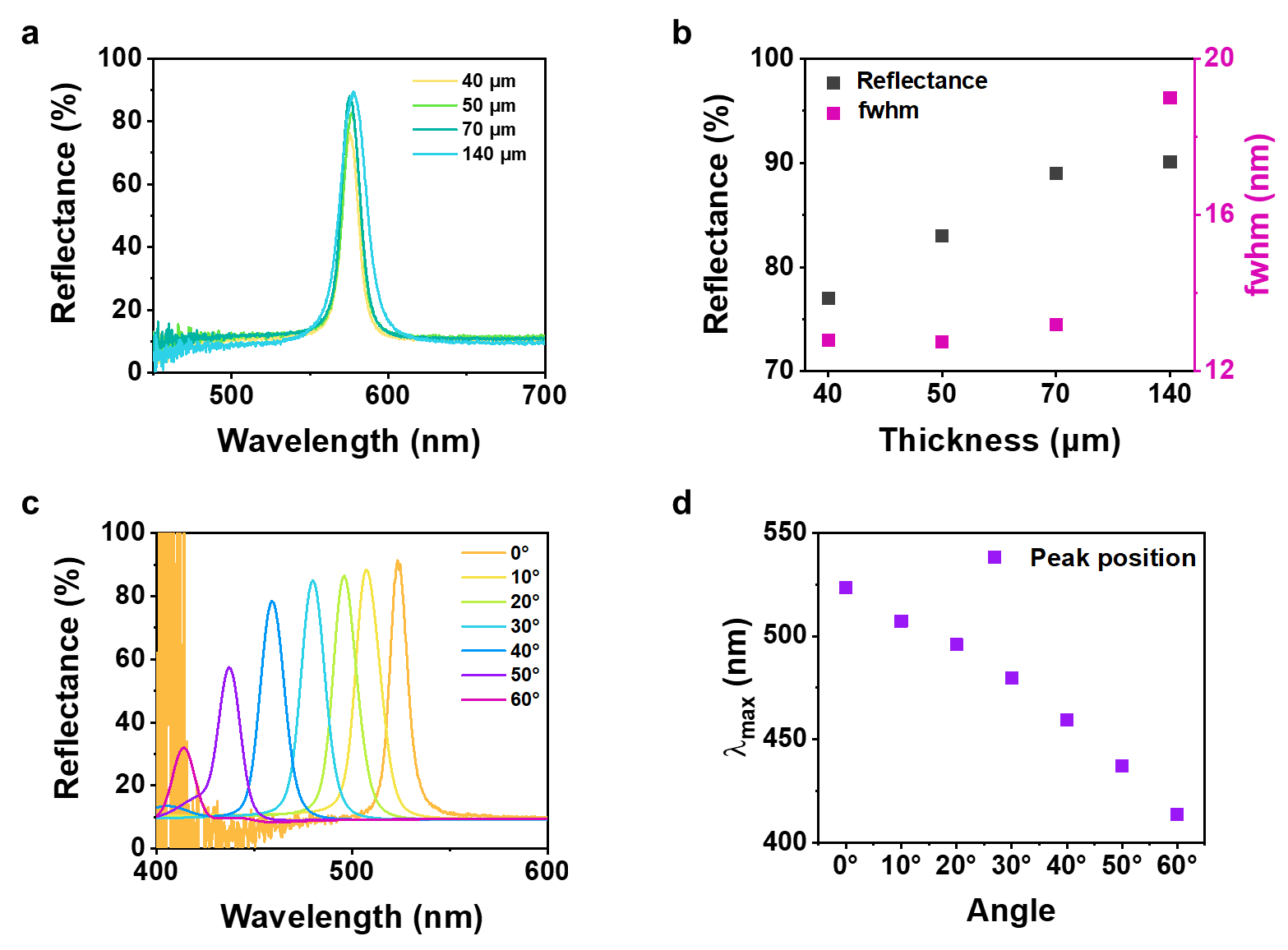
Fig. S11. Thickness-dependent reflectivity and angle-dependent reflectivity. a** Reflectance spectra of shear-applied photonic films with varied thickness. **b** Reflectance (left) and full width at half-maximum (right) of the photonic films with varied thickness. **c-d**  Reflectance spectra (c) and central wavelength at reflectance peak, *λ*_max_, (d) of shear-applied photonic films with various viewing angles.


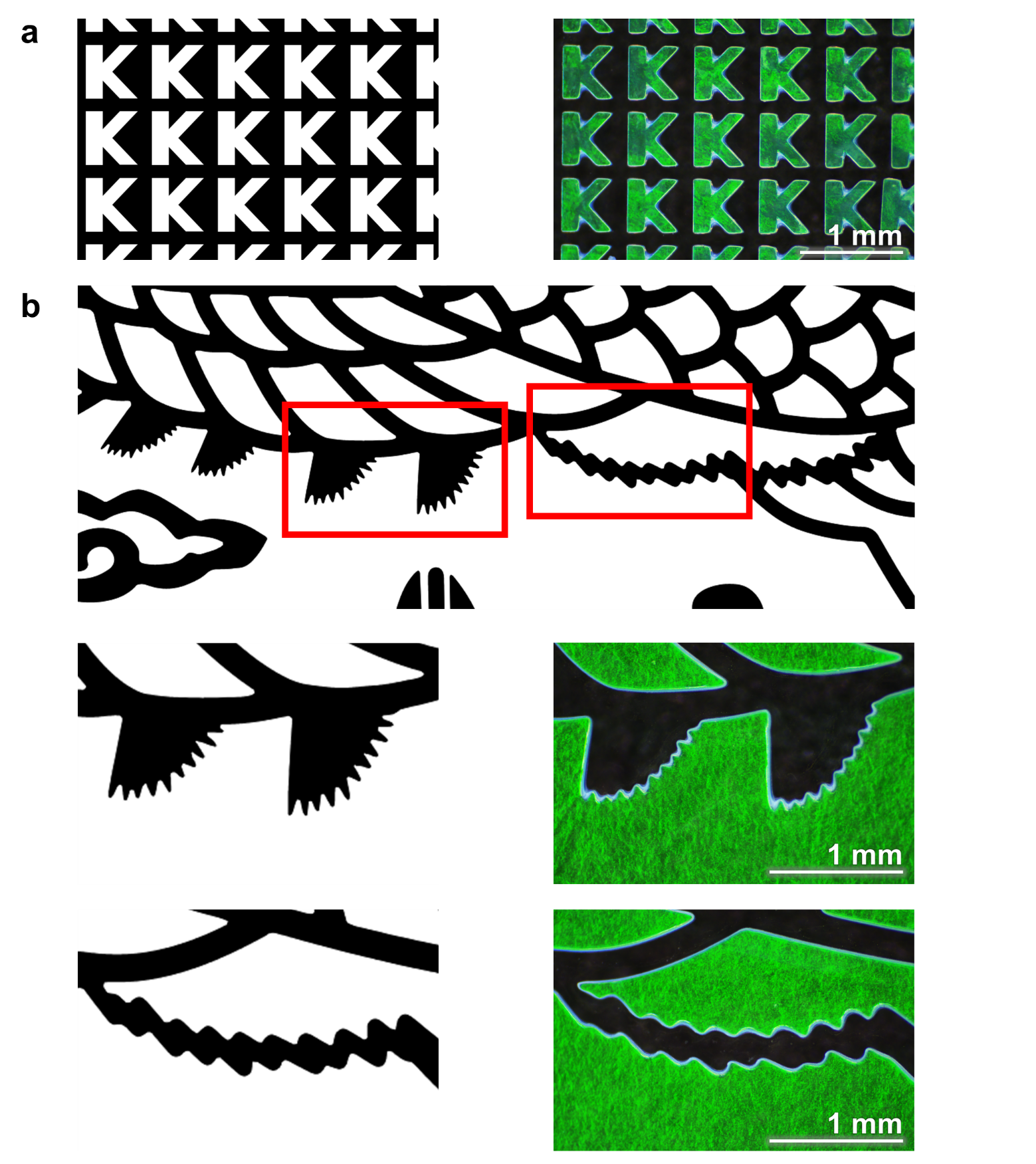


**Fig. S12. Resolution of Patterning. a** Set of photomask design and photograph of arrays of letter K. **b** Sets of photomask design and photographs of feather of cranes in intaglio. The magnified views show detailed features of the patterns (bottom right).


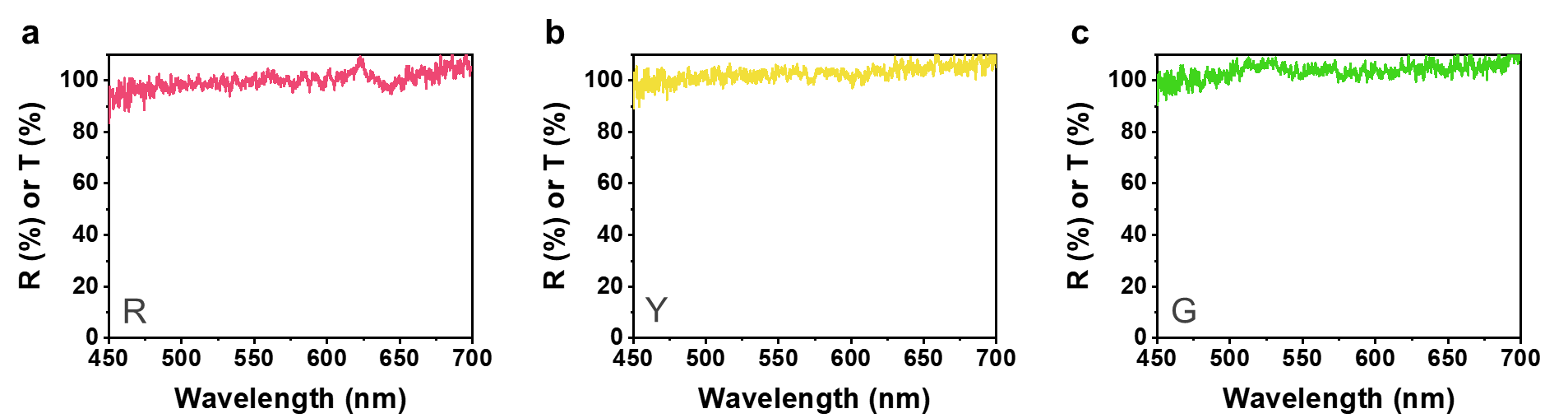


**Fig. S13. Sum of reflectance and transmittance. a-c** The sum of reflectance and transmittance of red leaf pattern (a), yellow leaf pattern (b) and green leaf pattern (c).


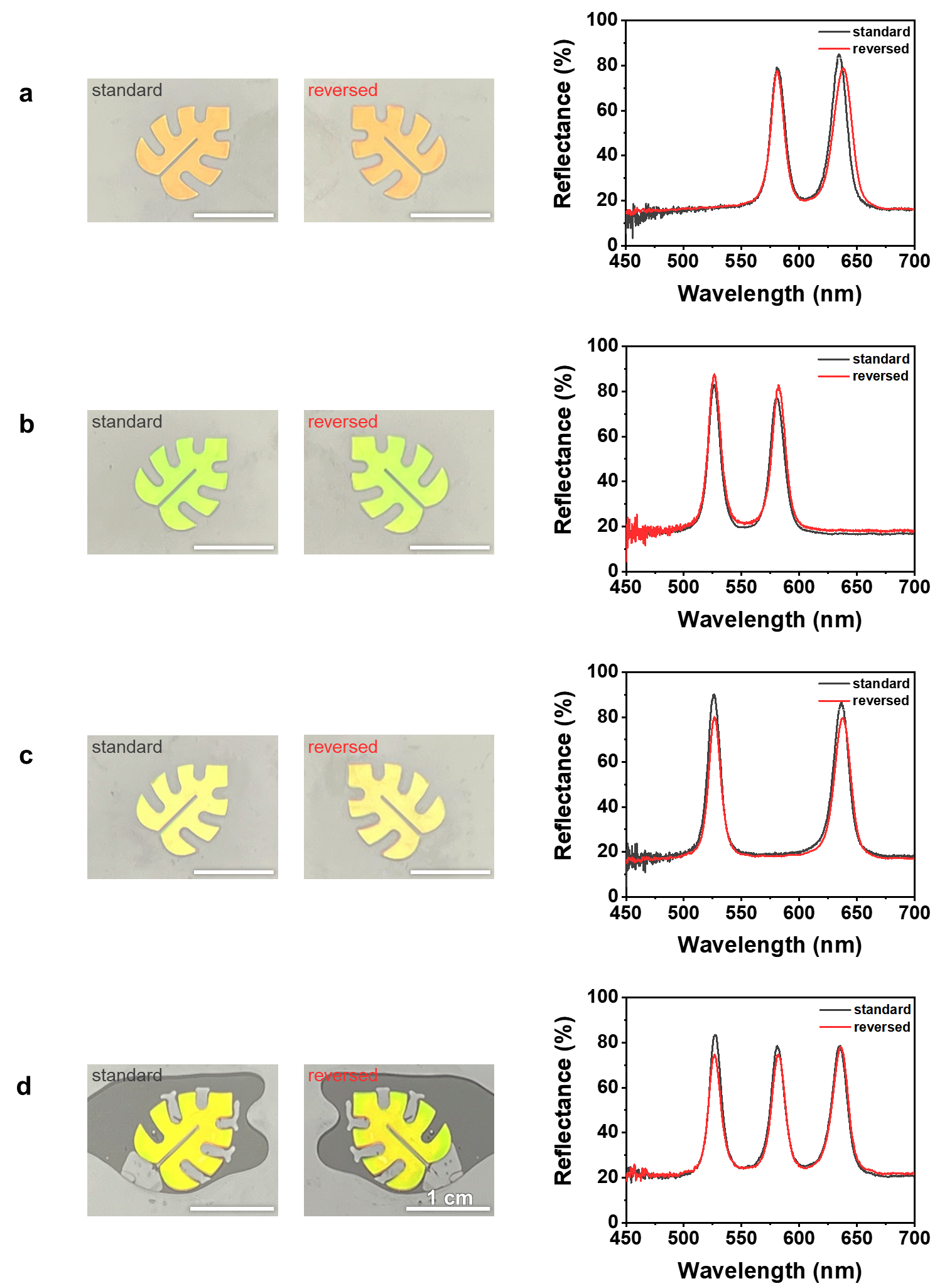


**Fig. S14. Comparison of multilayered photonic patterns on both sides.** Sets of photograph and reflectance in standard and reversed state of double-layered photonic leaf patterns **a** with combination of red and yellow, **b** combination of yellow and green, and **c** combination of red and green. **d** Same set for a triple-layered leaf pattern.
